# 2b or Not 2b: How Opposing FGF Receptor Splice Variants Are Blocking Progress in Precision Oncology

**DOI:** 10.1155/2021/9955456

**Published:** 2021-04-30

**Authors:** Richard J. Epstein, Li Jun Tian, Yan Fei Gu

**Affiliations:** ^1^New Hope Cancer Center, Beijing United Hospital, 9-11 Jiangtai West Rd, Chaoyang, Beijing 100015, China; ^2^Garvan Institute of Medical Research and UNSW Clinical School, 84 Victoria St, Darlinghurst 2010 Sydney, Australia

## Abstract

More than ten thousand peer-reviewed studies have assessed the role of fibroblast growth factors (FGFs) and their receptors (FGFRs) in cancer, but few patients have yet benefited from drugs targeting this molecular family. Strategizing how best to use FGFR-targeted drugs is complicated by multiple variables, including RNA splicing events that alter the affinity of ligands for FGFRs and hence change the outcomes of stromal-epithelial interactions. The effects of splicing are most relevant to FGFR2; expression of the FGFR2b splice isoform can restore apoptotic sensitivity to cancer cells, whereas switching to FGFR2c may drive tumor progression by triggering epithelial-mesenchymal transition. The differentiating and regulatory actions of wild-type FGFR2b contrast with the proliferative actions of FGFR1 and FGFR3, and may be converted to mitogenicity either by splice switching or by silencing of tumor suppressor genes such as CDH1 or PTEN. Exclusive use of small-molecule pan-FGFR inhibitors may thus cause nonselective blockade of FGFR2 isoforms with opposing actions, undermining the rationale of FGFR2 drug targeting. This splice-dependent ability of FGFR2 to switch between tumor-suppressing and -driving functions highlights an unmet oncologic need for isoform-specific drug targeting, e.g., by antibody inhibition of ligand-FGFR2c binding, as well as for more nuanced molecular pathology prediction of FGFR2 actions in different stromal-tumor contexts.

## 1. Introduction

Fibroblast growth factor receptors (FGFRs) are a family of transmembrane enzymes that coordinate ligand-dependent paracrine signaling between epithelial and stromal cells during embryonic development or adult adaptive responses [1–4]. Ten canonical secreted fibroblast growth factors (FGF1-10) activate four FGF receptor tyrosine kinases (FGFR1-4), and a dozen other FGFs comprise either circulating endocrine (e.g., FGF19) or nonsignaling intracellular (iFGF) peptides [5–7]; a fifth FGFR homolog lacking a catalytic domain acts as a ligand-sequestering decoy protein [8]. Binding of FGFs to heparan sulfate proteoglycans (HSPGs) and other noncanonical coreceptors in the extracellular matrix further complicates the *in vivo* dynamics of FGFR activation [9–11]. In addition, mutations affecting extracellular *N*-glycosylation sites can affect ligand binding and receptor function even when the amino acid substitution has no structural consequence [12]. FGF1- and FGF2-unglycosylated ligands, formerly termed acidic and basic FGF, activate numerous FGFR splice variants, whereas other FGFs tend to be more specific in their binding affinity. Ancient phylogenetic conservation of these diverse receptors and ligands implies important functional differences [13], precluding generalizations as to what constitutes prototypical FGF/FGFR actions across a vast spectrum of health and disease contexts [14].

Reflecting this complexity, the challenge of targeting a wide range of FGF-related genetic aberrations in human cancers [15–17] has proven more daunting than once hoped [18]. A key obstacle has been the rapid acquisition of drug resistance by tumors with FGFR aberrations [19], perhaps reflecting the high adaptivity of FGF signaling due to intra- and intercellular signaling fluxes [20–23], HSPG-ligand interplay [11], and splicing affecting any of the extracellular [24], kinase [25], juxtamembrane [26], or carboxyterminal [27] receptor domains. A related stumbling block has concerned frequent disconnects between FGFR-targetable genetic anomalies and their cellular; hence, therapeutic [28], sequelae [29]: for example, FGFR1-amplified squamous lung cancers [30] and FGFR3-mutant urothelial cancers [31], respond better than unselected cases to FGFR inhibitors, but survival benefits remain elusive [32, 33]. The finding that FGFR2-fused cholangiocarcinomas respond more durably is an advance [34], but resistance still hampers progress [35, 36]. These issues have led to FGFRs acquiring a problematic status in the personalised oncology space [37, 38], with FGFR defects arguably predicting drug resistance more reliably than survival gains [39–42].

Improving the value of FGFR-targeted anticancer treatments may therefore require closer attention to the underlying biology [43–45], including development of strategies to minimise the usual dose-limiting ocular and hyperphosphatemic toxicities of FGFR blockade [46]. To this end, the following perspective focuses on one such receptor, FGFR2, and its epithelial and mesenchymal splice isoforms, FGFR2b and FGFR2c.

## 2. Main Text

### 2.1. FGFR2 in Embryogenesis

The regulatory effects of FGFRs 1–3 are diversified by tissue-specific FGFR mRNA splicing events that determine ligand-binding specificity. This process is of most relevance to the FGFR2 gene which, unlike the other three FGFR genes, contains an open reading frame that has been invaded by large introns [47], implying a critical effect of splicing on transcriptomic regulation [48]. At least nine FGFR2 splice variants are reported, expression of which varies even within the same tumor type [24], while FGFR2 contains at least one and perhaps as many as three [49] more exons than other FGFRs [47]. Splicing of the FGFR2 third immunoglobulin-like domain produces either epithelium-specific IIIb (FGFR2-IIIb, i.e., FGFR2b, formerly keratinocyte growth factor receptor, KGFR/K-Sam) or mesenchymal IIIc (FGFR2c, Bek) receptors. FGFR2b is activated by mesenchymal FGF7 (keratinocyte growth factor, KGF1) or FGF10 (KGF2) [3] and also by FGF3 or FGF22, whereas mesenchymal FGFR2c responds to numerous ectodermal ligands including FGFs 2, 4, 6, 8, 9, 17, and 18.

#### 2.1.1. FGFR2 RNA Splicing in Morphogenetic Patterning

Splice switching between FGFR heteroisoforms is fundamental to normal development [50]. Two-way crosstalk between mesenchymal FGF ligands and epithelial FGFR2b balances the counteractivation of mesenchymal FGFR2c by epithelial FGFs, with such interactions underlying both physiological and pathological stromal-epithelial communication [51, 52]. Epithelial FGFR2b splice variants are expressed by ectodermal and endodermal tissues, including skin (keratinocytes, hair follicles, and sebocytes) and viscera (e.g., gut epithelium), in which context this isoform typically plays a prodifferentiating or -apoptotic role in response to FGF7/KGF or FGF10/KGF2 [53–57]. Knockout of the FGFR2b receptor or ligand expression leads to nonlethal congenital defects of hypospadias, anorectal malformations, glandular aplasia, or intestinal atresia [58–60], consistent with a role in epithelial patterning. In contrast, pan-*FGFR2* gene targeting manifests with lethal mesenchymal and bone defects, suggesting a more decisive role for FGFR2c than for FGFR2b in determining fetal viability [61].

#### 2.1.2. Germline FGFR2 Hyperactivation Syndromes

Illustrative of the morphologic effects of FGF signaling, the craniosynostoses are congenital syndromes in which constitutive kinase activity associated with FGFR2 missense mutations manifests with premature skull bone fusion, facial dysmorphism, cognitive dysfunction, and limb abnormalities. These phenotypes reflect accelerated mesenchymal apoptosis and/or differentiation [62], pathogenetically distinguishing them from the FGFR3-mutant germline ciliopathies, achondroplasia, and thanatophoric dysplasia [63].

The common craniosynostoses are Apert and Crouzon syndromes; the latter arises due to mutations causing overexpression of FGFR2c [64], consistent with its predominantly mesenchymal phenotype and its corresponding paucity of epithelial (e.g., skin) stigmata. In contrast, Apert-type FGFR2 mutations, two-thirds of which comprise Ser252Trp missense substitutions affecting the extracellular domain, extend the ligand responsiveness of FGFR2b to FGFR2c ligands [65, 66], causing a severe phenotype with combined epithelial and mesenchymal defects [67]. Premature osteogenic differentiation due to Apert mutations is blocked by soluble nonsignaling FGFR2 fragments containing the same mutation [68], confirming enhanced ligand affinity as the mechanism of receptor hyperactivation [69].

#### 2.1.3. FGFR2b-Dependent Phenotypes

The mesenchymal bone and cartilage stigmata of Apert syndrome arise via mutant *FGFR2*-induced upregulation of prodifferentiation p38-MAPK signaling [70]. However, the trademark epithelial presentation of this syndrome—extensive early-onset acne—is associated with induction of proinflammatory IL-1 further downstream [71, 72]. A direct genotype-phenotype relationship between acne and FGFR2 expression has been confirmed in postzygotic FGFR2 mosaics and in segmental acneiform nevi [73–75].

FGFR2b-activating ligands are inducible by the embryonic morphogen retinoic acid [76, 77] leading over time to FGFR2 downregulation [78]. It is via this pathway that retinoids, including the potent congener isotretinoin, improve both Apert and nonsyndromic acne [79, 80]. The same pharmacologic FGFR2-blocking mechanism likely causes the dysmorphogenetic teratogenicity of this drug, as well as of thalidomide [81].

### 2.2. Acne: A Clinical Window into FGFR2b Action

The FGFR2 inducibility of epithelial acne—whether sporadic (ligand-induced) or in Apert syndrome (constitutive)—is attributable to hyperactivation of FGFR2b [71]. Relevant to cancer control, acne is inducible not only by Apert-like mutagenic FGFR2 activation but also by epidermal growth factor receptor (EGFR) inactivation, either by homozygous knockout [82] or targeted drug inhibition [83]. This raises the hypothesis that FGFR2 plays a key intermediary role in both the skin toxicity and antitumor efficacy of EGFR-blocking drugs.

#### 2.2.1. FGFR2b-Inducible Acne following EGFR Signal Blockade

The folliculitis-like toxicities associated with EGFR blockade for diseases such as colorectal cancer (CRC) [83, 84] correlate with therapeutic efficacy, whereas tumoral resistance to these drugs is predicted by activating KRAS or BRAF mutations that make EGFR signaling redundant; in KRAS/BRAF-wild-type colon cancers, resistance to EGFR blockade may likewise arise from mutations upregulating FGFR2 function [85–87]. The targeted drug resistance associated in this context with FGFR2 overexpression is also predictive of poor tumor response to chemotherapy and radiotherapy [88], suggesting the concurrence of heterologous suppressor gene losses that impair apoptosis and thereby permit FGFR2 upregulation in these tumors (see below).

Pharmacologic EGFR inhibition in nonresistant CRCs is associated with compensatory FGFR2-induced p38/MAPK upregulation [72, 89] that induces the downstream proinflammatory mesenchymal mitogen IL-1*α* [90–94] which in turn promotes both sporadic acne [95] and iatrogenic folliculitis [96, 97]. Since IL-1 can also trigger cancer cell death [98, 99], the linked tumorilytic and acnegenic effects of EGFR blockade in such patients could reflect the same mechanism: namely, loss of negative feedback by (active) EGFR, leading by default to bypass upregulation of FGFR2b [100]. This bypass mechanism is reminiscent of c-RAF activation-induced skin toxicity that occurs when melanoma patients receive BRAF inhibitors [101]. Consistent with this, prevention of EGFR inhibitor-induced acne by skin irradiation is attributable to FGFR2 downregulation [102]; FGF7-activated FGFR2b causes TGF*α*-induced EGFR signaling [103], completing a homeostatic FGFR2-EGFR control loop. Drug inhibition of EGFR signaling may thus release a feedback constraint on FGFR2b which manifests off-target as IL1-mediated skin toxicity [71], predicting on-target tumor response.

A key nexus for FGF and EGF signaling is the phosphotyrosine-independent binding of the adaptor protein FRS2*α* to FGFR juxtamembrane sequences via its PTB domain [104]. Ligand-dependent FGFR activation causes FRS2*α* tyrosine-196 phosphorylation which triggers SH2 domain binding of Grb2 (or the tyrosine phosphatase SHP2), activating the mitogenic Ras-Raf-MEK-ERK (p42-MAPK) pathway [105] and driving proliferation of mesenchymal tissues expressing FGFR2c [106, 107]. EGF abrogates FGF-inducible FRS2*α* phosphorylation in an ERK-dependent manner [108], implicating FRS2*α* as a negative regulatory node in this EGF/FGF signaling network [109].

#### 2.2.2. FGFR2b as a Mediator of Androgenic Acne and Alopecia

The causes of FGFR2b-induced acne are not only genetic or iatrogenic but also androgenic. Expression of FGFR2b-specific ligands FGF7 [110] and FGF10 [59] is androgen dependent, with androgen-induced upregulation of FGF10 [111] being implicated in the pathogenesis of adolescent acne [79]. In the rare seborrhea-acne-hirsutism-alopecia (SAHA) syndrome [112], acne responds equally well to isotretinoin (which downregulates FGFR2b) and antiandrogens (which block transactivation of FGF7/10) [113]. This latter syndromic association between androgens, FGFR2, acne, and alopecia raises the intriguing hypothesis that in addition to FGFR2b-induced inflammatory folliculitis, androgen-dependent FGFR2b proapoptotic signaling could be involved in the pathogenesis of male-pattern baldness.

Consistent with this, dominant-negative EGFR-silenced transgenic mice exhibit striking hair follicle necrosis due to failure of catagen entry [114]. Since EGFR drives follicle proliferation and blocks differentiation [82] whereas FGFR2b promotes follicle differentiation [115, 116], increased androgen-inducible FGFR2b signaling in the presence of sustained EGF exposure [117] could trigger follicle autophagy and hair loss, raising novel treatment strategies. Indeed, both FGF7 [118–120] and FGF10 [121] are implicated as driving telogen/anagen transition during hair growth *in vivo*, though whether this is accompanied by FGFR2b downregulation (vs. sustained activation) remains unclear. These observations highlight that FGFR2 action is best understood in the context of ambient signaling by other hormones and cytokines, and suggest how FGFR2b-specific drug inhibitors—whether systemic or topical (e.g., photodynamic therapy [122])—could improve therapy of nonmalignant diseases.

### 2.3. FGFR2b in Tumor Suppression

Common hardwired cancer-associated FGFR genotypes include FGFR1 amplification (e.g., in lung cancers, uveal melanomas, low-grade gliomas, or drug-refractory breast cancers [39, 123, 124]), FGFR3 mutations (e.g., in superficial papillary urothelial cancers [125, 126]), and FGFR2 gene fusions (especially in fluke-negative intrahepatic cholangiocarcinomas [127]). Unlike FGFR1 and FGFR3—firmly implicated as oncogenic drivers in tumor invasion and proliferation [128–130]—wild-type FGFR2b often acts in a tumor-suppressive manner to promote differentiation or apoptosis [131–136], with FGFR2b downregulation mirroring FGFR1 upregulation during progression or epithelial-mesenchymal transition (EMT) [137–142]. Restoration of FGFR2b signaling may also enhance the chemosensitivity [143] and radiosensitivity [144] of treatment-refractory cancer cells by lowering apoptotic threshold.

#### 2.3.1. Regulatory Functions of FGFR2b

The *FGFR2* gene is structurally distinguished among FGFRs by having a promoter CpG island enabling epigenetic transrepression, e.g., in neoplasms of the pituitary [145, 146] or bladder [131]. Methylation-induced transcriptional FGFR2 downregulation is thus associated with poorer prognosis in hepatocellular carcinoma [147] and bladder cancer [131], analogous to functional losses caused by FGFR2 deletion in glioblastomas [148], or loss of FGFR2 heterozygosity in osteosarcomas [149].

Conversely, immunohistochemical FGFR2b expression is positively associated with well-differentiated morphology and inversely with Ki-67 in normal colorectal mucosa and CRCs [150]. The prodifferentiation and proapoptotic effects of FGFR2b [151, 152] have been linked to certain effector steps, most notably including autophagy mediated by JNK1 [153, 154]. Additional downstream pathways in this tumor-suppressive context include Cbl-dependent ubiquitin downregulation of Lyn and Fyn kinases [155] and/or bystander effects on FGFR3-expressing stromal cells [156].

#### 2.3.2. Functional Alterations due to FGFR2 Gene Splicing

Splice-dependent insertion of a juxtamembrane domain VT (valine-threonine; VT+) dipeptide enables FRS2*α* binding, enhancing Raf-ERK signaling [26]. Epithelial FGFR2b with splice-dependent loss of this dipeptide (VT−) would thus be predicted to lose mitogenic Grb2-Ras-Raf-ERK signaling [157] but not C-terminal Tyr-766 PLC-*γ* activation or proinflammatory/-apoptotic p38-MAPK/IL-1 and JNK signaling. Alternatively, FGFR2b receptors could be converted to oncogenicity by C-terminal truncations that abolish Tyr-770 PI3K docking [27], leading to faster receptor internalisation and upregulated FRS2*α* signaling [158].

Spliceosomal dysregulation may switch FGFR2b to FGFR2c, altering ligand binding and driving dedifferentiation [159] or EMT [160], e.g., during progression of hormone-dependent to -resistant cancers [161, 162], or associated with constitutive EGFR activation in HPV16 E5-induced neoplasia [163]. The more tumorigenic role of FGFR2c relative to FGFR2b [164] is clear, with FGFR2c overexpression correlating with tumor growth and metastasis [52, 133, 165, 166]. In contrast, the relationship of FGFR2b overexpression to vascular invasion, for example, is reported as positive in some clinical contexts [167] and negative in others [147], suggesting unmeasured confounding variables. Splice switching from FGFR2b to FGFR2c has been reported in 90% of clear-cell renal carcinomas, whereas less-aggressive lesions such as oncocytomas continue to express FGFR2b [159].

### 2.4. FGFR2b in Oncogenesis

Activating FGFR2 missense mutations or amplifications can occur in cancers of the stomach, breast, lung, endometrium, and ovary [168], and overexpression of the FGFR2b-specific ligand FGF10 is linked to worse prognosis in breast cancer [169]. However, cancer occurrences in the mutant-activated FGFR2 craniosynostoses appear confined to rare cases associated with chromosomal losses [170]. This discrepancy suggests that FGFR2-mutant syndromes lack heterologous regulatory defects in the germline akin to those that permit uncontrolled FGFR2 activation in somatic tumors [171, 172], such as TP53 loss [173].

#### 2.4.1. FGFR2 in Carcinogenesis Associated with Inflammatory Bowel Disease

Relevant to this, FGFR2 may play a causal role in the natural history of CRCs arising from inflammatory bowel disease (IBD): the p38 pathway downstream of FGFR2b drives the chronic inflammatory phenotypes of ulcerative colitis and Crohn's disease [174, 175], with colitis-associated tumors characterised by gain-of-function FGFR2 aberrations [172, 176] such as have been reported in fewer than 2% of sporadic CRC [177]. Furthermore, activating FGFR2 mutations occur in nondysplastic colorectal mucosa from ulcerative colitis patients, together with defects of the E-cadherin-encoding suppressor gene CDH1 [178]. Colonic inflammation mediated by overexpression of proapoptotic FGFR2 may thus select for loss-of-function CDH1 defects, in turn permitting mutagenic fixation of FGFR2 hyperactivation.

#### 2.4.2. FGFR2b and E-Cadherin/CDH1 in Diffuse Gastric Cancers

The proapoptotic effects of normal ligand-inducible FGFR2 activation depend on cadherin-dependent epithelial cell adhesion [179]. FGFR2b signaling may induce p38-dependent expression of CDH1 and its product E-cadherin, leading to tumor suppression [180]. Splice switching from FGFR2b to FGFR2c reverses these effects via alternative upregulation of the stromal adhesion molecule N-cadherin, with this FGFR2b-downregulating splicing change contributing to loss of E-cadherin expression and EMT [160, 181].

In contrast, tumors driven by amplified FGFR2 in which wild-type FGFR2b is the overexpressed isoform [182] tend to occur within tissues that are E-cadherin-deficient. The prototype example is diffuse gastric cancer defined by CDH1 gene loss [183, 184], which suggests that lack of E-cadherin enables FGFR2b to drive cell growth and invasion without apoptosis [185]. Small-molecule FGFR2 inhibitors may temporarily control such E-cadherin-deficient cancers [186] due to nonselective FGFR2c cross-inhibition; however, such treatments also select for EMT-driven drug resistance [187]. In gastric cancers already associated with EMT, TWIST repressor protein upregulation leads to further E-cadherin downregulation [188], accelerating epithelial dedifferentiation, FGFR2c overexpression, and tissue invasion [189].

#### 2.4.3. FGFR2b and PTEN in Endometrial Cancers

Acquired genetic changes disrupting the PTEN tumor suppressor pathway represent another frequent cancer-associated source of apoptotic resistance. Such defects often occur in low-immunogenicity hormone-driven tumors such as breast and prostate cancer [190] and permit overexpression of the PIK3CA gene which in turn activates antiapoptotic signaling via the phosphatidylinositol-3'kinase (PI3K)-AKT-mTOR pathway [191]. In postpubertal females, proapoptotic signaling is central to hormone-dependent endometrial deciduation during menstruation; consistent with this, another hormonally induced human tumor subtype—endometrioid endometrial cancers—exhibits PTEN loss and/or PI3K oversignaling in 90% of cases, often associated with Apert-like FGFR2 mutations [135, 192] that upregulate FGFR2c [193]. The idea that increased FGFR2 signaling confers a growth advantage which depends on PTEN-defective apoptotic defects is supported by experiments showing that coinhibition of FGFR2 [194] and PI3K [195] restores growth control. Overexpression of EMT-promoting FGFR2c [133] confers a worse prognosis in PTEN-defective endometrial carcinoma [196], whereas non-endometrioid endometrial cancers with wild-type PTEN lose E-cadherin expression en route to EMT [197]. These disparate tumor trajectories show how the proapoptotic function of FGFR2 may be converted to tumorigenicity by a variety of epistatic suppressor gene defects [135].

Relevant to brain tumors, the 10q26.13 FGFR2 chromosomal locus is sited adjacent to the WDR11 tumor suppressor gene [198] near the PTEN deletion site at 10q23.31 and the methylatable (repressible) MGMT suppressor gene promoter at 10q26.3 [199] which predicts chemotherapy response and natural history [200–202]. Deletion of PTEN leads to mTORC1-induced upregulation of the FGFR2b ligand FGF10, promoting tumor outgrowth [203]. PTEN deletion may also trigger upregulation of the FGFR2c ligand FGF18 and downregulation of FGFR2 repressor SPRY2 [204]. FGFR2 actions thus vary as a dynamic function of PTEN pathway signaling integrity, in some ways paralleling the above relationship with CDH1/E-cadherin.

#### 2.4.4. FGFR2b and ΔNp63*α* in Squamous and Colorectal Cancers

The p53 family of stress-responsive cell-cycle control proteins includes p63 and p73. Like FGFR2, the TP63 gene family plays key roles in craniofacial morphogenesis [205], skin differentiation, and apoptosis [206]. Epithelial barrier integrity is dependent upon the expression of wild-type p63 [207] which in turn regulates epithelial-mesenchymal crosstalk via induction of FGFR2b [208]. A mechanistic link between these pathways is supported by molecular correlates of AEC (ankyloblepharon-ectodermal-clefting, Hay–Wells) syndrome, in which germline loss-of-function TP63 mutations are associated with pathologically high FGFR2b expression accompanied by failure of epithelial differentiation [209].

Like FGFR2, several TP63 gene isoforms (*α*, *β*, and *γ*) are regulated by differential splicing. Two p63*α* isoforms are transcribed from different promoters: a proapoptotic transcriptionally activating (TA) isoform that includes an *N*-terminal transactivation domain, called TAp63*α*, which is the main p63 isoform expressed in normal colon and overexpressed in CRC [210]—in which context it exerts a tumor-suppressive effect [211]—and a dominant-negative p53-inhibitory *N*-terminal-truncated (ΔN) isoform lacking the transactivation domain, called ΔNp63*α*, which is the main isoform in keratinocytes [212]. Activating FGFR2 mutations or amplifications [213] or FGFR2b ligand overexpression [214] often occur in tumors lacking TAp63 protein due to ΔNp63 substitution [206, 215], particularly squamous cancers [210, 216, 217]. Like FGFR2c [52] but not FGFR2b [54], ΔNp63*α* suppresses JNK-dependent differentiation and apoptosis in an ERK-dependent manner [218], and its overexpression confers a poor prognosis upon urothelial cancers [219] which release exosomes that trigger EMT in adjacent urothelium [220], creating positive feedback between FGFR2c and ΔNp63*α* [52, 216]. In the colon, TAp63*α* represses EGFR expression, leading to feedback upregulation of proapoptotic TP53 that normally transactivates the EGFR gene via a promoter response element lacking in TP63 [221]. Anticancer drug-induced EGFR kinase silencing may thus cause CRC response via a compensatory FGFR2b-mediated JNK1 and IL-1 signal which occurs against a background of p53 upregulation and TAp63*α* expression.

### 2.5. FGFR2 in Anticancer Therapy

Amongst the ligand-inducible FGFRs, only FGFR4— genetic aberrations of which, compared with FGFRs 1–3, are seldom implicated as tumorigenic drivers—has sufficient divergence in its kinase structure to enable selective small-molecule inhibition [222], e.g., by drugs such as BLU9931 [223]. Since homozygous FGFR4 gene knockout causes no phenotypic loss of viability or fertility [224], this gene may have regulatory (tumor-suppressor) rather than mitogenic (oncogenic) properties [225]. For oncologic purposes, then, drugs inhibiting FGFRs 1–3 may be considered “pan”-FGFR inhibitors, and many inhibitors so defined—including TAS-120 [226], AZD4547, CPL304110 [227], BGJ398 (infigratinib) [228], and Debio 1347 [229]—have already been tested in trials [230]. The term “receptor nonselectivity” may therefore be better applied to drugs that cross-inhibit multiple receptor tyrosine kinases, such as VEGFR2, PDGFR*β*, CSF1R, FLT3, and KIT, and which include broad-spectrum partial FGFR antagonists such as dovitinib, ponatinib, and lenvatinib [38].

#### 2.5.1. Clinically Effective FGFR Inhibitors

Two drugs have so far been FDA-approved for tumors expressing targetable genetic aberrations of FGF signaling, and both are small-molecule pan-FGFR inhibitors. The first is erdafitinib, which is licensed for patients with advanced bladder cancers expressing either FGFR3 or FGFR2 aberrations [231]. A phase II trial reported response rates of 40% and progression-free survival of 5.5 months, with best and worst response rates in FGFR3-mutant vs. FGFR2/3-fused bladder tumors, respectively [31, 232]. Adverse events were frequent, often grade 3 or higher, and mandated close monitoring [233]. Despite this promising evidence of efficacy in trials [234], immunotherapies have to date largely outcompeted FGFR-based approaches for second-line palliation of platinum-refractory urothelial cancer [235].

For unresectable intrahepatic cholangiocarcinomas expressing FGFR2 gene fusions or similar rearrangements, the FDA has approved pemigatinib, an FGFR1-3 inhibitor earlier approved for orphan treatment of hematologic malignancies with PDGFR or JAK2 aberrations [236]. In FGFR2-fused or -rearranged tumors, 35–40% responded to pemigatinib; however, all tumors with nonfusion FGFR2 mutations or copy-number variations failed to respond, as did all tumors with FRS2 amplification. Furthermore, even among those FGFR2-fused tumors responding to pemigatinib, most acquired resistance-conferring mutations [237, 238]. These results imply that responses to broad-spectrum FGFR-inhibitory monotherapies require powerful FGFR2 signaling agonism [239]—as predicted by fusions causing FGFR2 dimerization [240]—but that this might still not translate into substantive real-world benefit.

#### 2.5.2. Strategies to Improve Patient Benefit

Given the above evidence for tumor-suppressive activity of FGFR2b, one key strategic concern should be that empirical use of small-molecule pan-FGFR kinase antagonists cross-inhibiting multiple receptors [241] may directly select for resistance. Primary resistance may be predetermined in some cases by de novo regulatory defects affecting proapoptotic genes such as TP53 or CDKN2A; acquired resistance to FGFR-targeted drug therapies may also supervene via selectable changes, including not only new gain-of-function FGFR mutations [35] but also transmodulatory crosstalk [23, 242], targeting errors due to splice-dependent signaling variations [243, 244], and/or multistep loss-of-function tumor suppressor pathway derangements such as those affecting CDH1 or PTEN. Since knockdown of wild-type FGFR2b promotes cell dedifferentiation [54, 245], drugs including this isoform in their inhibitory spectrum could accelerate resistance [181].

More customised approaches to the dose-limiting toxicities of FGF(R)-targeted drugs should improve therapeutic ratios. A distinctive toxicity of this drug class is hyperphosphatemia due to increased renal tubular phosphate reabsorption, which affected 70% of patients in trials of small-molecule FGFR inhibitors [46]. Since hypophosphatemia is induced by FGF23 but not by other ligands for FGFR1 [246]—the main phosphate-regulating FGFR—cotreatment with recombinant FGF23 is one approach that could, in theory, mitigate this toxicity.

This illustrates that one strategy for improving the therapeutic index of FGF(R)-targeted drugs is combination therapies [247–249], i.e., network-based regimens that reflect the interactive mesh-like regulation of FGF signaling [45, 250]. For example, missense mutations causing combined activation of FGFR2 and VEGFR2 occur together in a subset of CRCs [177], raising the possibility of dual therapy. On a cautionary note, however, relatively few combinations of targeted drugs with other medications have so far proven to be game-changers.

#### 2.5.3. Better Personalisation of FGFR2-Targeted Therapies

An ideal dataset to predict FGFR2-targeted drug success would include not only the specific actionable FGFR2-related aberration (e.g., fusion, missense mutation, and amplification) but also quantification of the pattern of FGFR2b vs. FGFR2c expression by the tumor itself; pathway fingerprinting of other nontargeted receptor tyrosine kinase (e.g., EGFR, VEGFR, or other FGFRs) signaling, whether up- or downregulated, within the tumor and stroma, e.g., by using phosphoantibody profiling; and tumor suppressor gene aberrations implicated in either FGFR2 function switching (such as CDH1, PTEN, and TP63; see above) or disease prognosis (e.g., TP53 or CDKN2A [237]).

Strategies beyond small-molecule pan-FGFR inhibitors are needed [18, 251], and newer approaches offer hope [28, 252]. The importance of separately targeting FGFR2b and FGFR2c has been recognized by researchers for over a decade [253]. An example of isoform-specific FGF signaling inhibition is bemarituzumab (FPA144), a monoclonal antibody that targets FGFR2b and prevents ligand binding; either FGFR2b immunohistochemistry or FGFR2 amplification by ctDNA has been used for patient selection and trial eligibility [254, 255]. Antibodies specific for the extracellular domain of FGFR2 have also been synthesised—not for blocking isoform-specific ligand binding, but rather for attaching cytotoxic moieties and thus targeting FGFR2-overexpressing tumors [256]. Although such an approach could prove effective in some contexts, e.g., FGFR2 amplification [257], it does not address the issue of differential FGFR2 isoform coexpression in tumors lacking such amplification.

Ligand-specific antibodies—to FGF8 and FGF2, though not yet to more specific FGFR2 growth factors such as FGF7 or FGF10—have also been tested. Another strategy is to use FGFR isoform-specific extracellular domains as low-toxicity decoys or “ligand traps” for growth factors [258, 259], recapitulating the physiological inhibition of FGFR2c by FGF2-binding (but kinase-inactive) FGFR5 [8]. The prospect of FGFR2c-specific antibodies selectively blocking extracellular ligand binding would seem likely to have wide-scale therapeutic applications [166]. One concern about such high-precision signal-inhibitory treatments is whether they will yield sufficient benefit when used as monotherapies in early-phase clinical trials—which outcome might not be rationally expected, given the network-based signaling involved—to justify subsequent inclusion in the combination protocols that seem most likely to transform patient outcomes.

## 3. Conclusions

FGFR2 differs from its homologs FGFR1 and FGFR3 in terms of epigenetic repressibility, structural predisposition to splicing-dependent variants, and androgen-dependent inducibility of its high-specificity ligands. The FGFR2b isoform also differs markedly from its splice partner FGFR2c in terms of its prodifferentiation and proapoptotic functions, which can be abrogated by heterologous suppressor gene aberrations. These differences underlie a growing need for today's oncologists and trialists to understand the biological spectrum of FGFRs and hence to factor in more sophisticated predictive data for stratifying patients and tumors.

These concerns are relevant to future anticancer drug development (see Table 1). Pan-FGFR druggability strategies remain in vogue as a way of circumventing the association of FGFR gene aberrations with resistance, but this approach faces limitations which now look likely to defy empirical research. The adaptive complexity of FGF signaling has created a moving target of unprecedented difficulty for the young science of precision oncology, but biomarker-informed targeting using isoform-specific large molecules that block either FGFR2c or FGFR2b/FGF7 could prove a useful starting point for the next phase of this key therapeutic challenge.

## Figures and Tables

**Table 1:** Key findings from this analysis.



1	The FGFR2 gene undergoes differential splicing in normal epithelial and mesenchymal tissues, resulting in respective FGFR2b and FGFR2c receptor splice isoforms that differ in the affinity of their extracellular domains for specific ligands.

2	The FGFR2b receptor isoform often exerts prodifferentiation and proapoptotic effects, whereas the FGFR2c isoform is promitogenic. Pathological tumor splice switching from FGFR2b to FGFR2b is causally implicated in the mechanism of tumor progression via epithelial-mesenchymal transition (EMT).

3	In the small subset of tumors that overexpress wild-type FGFR2b, heterologous variants of regulatory genes such as *CDH1*, *PTEN*, or *TP63* are often coexpressed. This implies that interpreting the actionable tumor genotype may require more inclusive consideration of nonactionable genetic aberrations.

4	Small-molecule kinase-inhibitory drugs that simultaneously cross-inhibit FGFR2b and FGFR2c could have unintended and/or conflicting effects in tumor or stromal compartments.

5	Clinically effective tumor-specific FGFR2 drug targeting will depend in part on greater use of nuanced diagnostic assays that clarify which receptor isoform is driving tumor behavior within any unique epistatic context of genetic changes.

6	New FGFR2 isoform-specific drugs are now available for trial use. Their long-term success is likely to be determined in part by the molecular sophistication of patient/cancer selection and by their rational use in combination with other targeted drugs.

## Abbreviations

CRC: Colorectal cancer
EGF(R): Epidermal growth factor (receptor)
EMT: Epithelial-mesenchymal transition
FGF(R): Fibroblast growth factor (receptor)
HSPG: Heparan sulfate proteoglycan
IBD: Inflammatory bowel disease
IL-1: Interleukin-1
KGF(R): Keratinocyte growth factor (receptor)
PI3K: Phosphatidylinositol-3'-kinase
SAHA: Seborrhea-acne-hirsutism-alopecia (syndrome)
TA: Transactivating
TGF*α*: Transforming growth factor-alpha
VEGF: Vasoactive endothelial growth factor.
